# Retaining interest in caring for underserved patients among future medicine subspecialists: Underserved Medicine and Public Health (UMPH) program

**DOI:** 10.1186/s12909-021-03006-x

**Published:** 2021-11-20

**Authors:** Jillian S. Catalanotti, David K. Popiel, April Barbour

**Affiliations:** 1 Department of Medicine, The George Washington University, Washington, DC, USA; 2 Department of Emergency Medicine, The George Washington University, Washington, DC, USA

**Keywords:** Underserved medicine, Community health, Graduate medical education

## Abstract

**Background:**

Accessing subspecialty care is hard for underserved patients in the U.S. Published curricula in underserved medicine for Internal Medicine residents target future-primary care physicians, with unknown impact on future medicine subspecialists.

**Methods:**

The aim was to retain interest in caring for underserved patients among Internal Medicine residents who plan for subspecialist careers at an urban university hospital. The two-year Underserved Medicine and Public Health (UMPH) program features community-based clinics, evening seminars, reflection assignments and practicum projects for 3–7 Internal Medicine residents per year. All may apply regardless of anticipated career plans after residency. Seven years of graduates were surveyed. Data were analyzed using descriptive statistics.

**Results:**

According to respondents, UMPH provided a meaningful forum to discuss important issues in underserved medicine, fostered interest in treating underserved populations and provided a sense of belonging to a community of providers committed to underserved medicine. After residency, 48% of UMPH graduates pursued subspecialty training and 34% practiced hospitalist medicine. 65% of respondents disagreed that “UMPH made me more likely to practice primary care” and 59% agreed “UMPH should target residents pursuing subpecialty careers.”

**Conclusions:**

A curriculum in underserved medicine can retain interest in caring for underserved patients among future-medicine subspecialists. Lessons learned include [1] building relationships with local community health centers and community-practicing physicians was important for success and [2] thoughtful scheduling promoted high resident attendance at program events and avoided detracting from other activities required during residency for subspecialist career paths. We hope Internal Medicine residency programs consider training in underserved medicine for all trainees. Future work should investigate sustainability, whether training results in improved subspecialty access, and whether subspecialists face unique barriers caring for underserved patients. Future curricula should include advocacy skills to target systemic barriers.

## Background

Health disparities have numerous causes, among them disparate access to primary care and to timely subspecialty care. In 2018, the U.S.’s 7015 Health Professional Shortage Areas (HPSAs) were home to over 78 million people [1]. In the U.S., medical care must be purchased, most commonly by a health insurance plan and/or an individual. Physicians are not legally required to provide non-emergency care, nor to accept any particular health insurance plan. Low reimbursement rates compared to other insurance plans are the leading reason physicians do not accept Medicaid, the government-funded health insurance plan for qualified poor Americans [2]. According to a 2015 survey of outpatient physicians across multiple fields, 71% accepted Medicaid, 85% accepted Medicare and 90% accepted private insurance [3].

The Affordable Care Act expanded the number of patients who qualified for Medicaid, markedly decreasing the number of uninsured patients, but made no provisions to address the resultant increased demand for subspecialty care [4]. The Association of American Medical Colleges projects a potential national shortage of up to 49,300 primary care physicians (PCPs) and up to 9600 medicine subspecialty physicians by 2030 [5]. Demographic trends in the U.S. suggest that the overall demand for physicians is likely to grow proportionally faster for minority populations [6].

Federally Qualified Health Centers (FQHCs) provide primary care medical services to patients from underserved populations. In exchange for funding from the federal government, FQHCs must see all patients regardless of ability to pay or health insurance status. In a 2004 survey of medical directors of FQHCs by Cook, et al.*,* respondents reported that 25% of visits resulted in referrals to medical services not provided at the center [7]. These subspecialty services were harder to obtain for patients with Medicaid and markedly more challenging for uninsured patients [7]. The most frequently reported barriers to access were providers not accepting patients’ insurance, requiring payment up front, and insurance not covering the services requested. Two of these three barriers are provider- or practice-dependent. In a 2001 survey of medical directors of community health centers (CHCs), 35% of respondents said they or their physicians attempt to negotiate with off-site subspecialists on behalf of uninsured patients to obtain lower rates, and 20% reported that physicians rely upon professional networks and friends to provide subspecialty care to uninsured patients, a practice informally known as “tin cup medicine.” [6, 8]

Data from the Community Tracking Study Physician Survey showed that from 1996 to 2005, both the number of physicians providing charity care (i.e., care for which no pay is sought), and the number seeing Medicaid patients decreased [9]. Medical directors of FQHCs affiliated with a medical school or hospital reported less difficulty accessing subspecialty care [7], however in a 2003 survey of faculty at 121 academic health centers, “at least one faculty member from [96%] of the institutions reported they were discouraged from seeing indigent patients.” [10]

Although the National Health Service Corps and state-level loan-repayment programs aim to recruit physicians into primary care in shortage areas, physicians in medicine subspecialties have limited external incentives to provide care to underserved patients. Likewise, Title VII grants aim to increase the training of future PCPs with priority given to those residency programs that demonstrate success in placing graduates in HPSAs, but there is no federal program to incentivize training future subspecialists to meet the medical needs of these same patients.

Programs and policies to support subspecialists to pursue careers that include caring for patients from underserved populations are needed. Because some barriers to accessing subspecialty care are provider- or practice-dependent, producing subspecialty physicians with the beliefs, knowledge and skills to care for underserved patients is imperative.

**Fig. 1:**
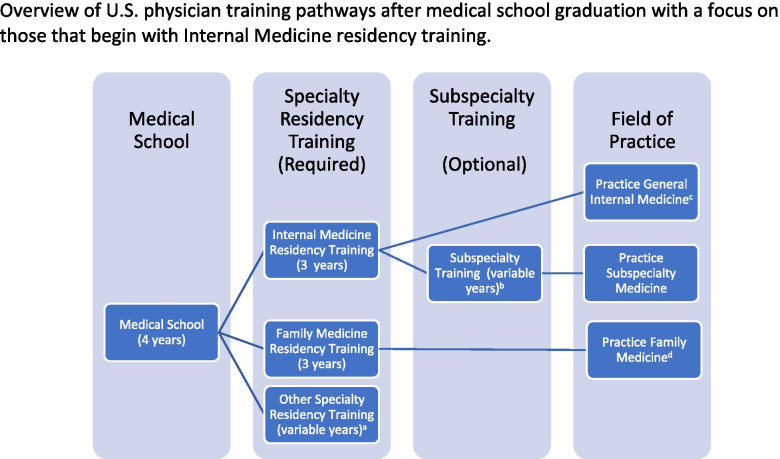
Overview of U.S. physician training pathways after medical school graduation with a focus on those that begin with Internal Medicine residency training. **a** An extensive list of other specialty residency training paths is outside the scope of this paper. **b** The most common subspecialties of Internal Medicine in the U.S. are Allergy/Immunology, Cardiology, Endocrinology, Gastroenterology, Geriatrics, Hematology & Oncology, Hospice & Palliative Medicine, Infectious Diseases, Nephrology, Pulmonology & Critical Care Medicine, and Rheumatology. **c** May practice outpatient general Internal Medicine (commonly called primary care), inpatient general internal medicine (called hospitalist medicine), or a combination of both. **d** A limited number of clinical subspecialty training options exist after Family Medicine residency training, however most of these physicians practice outpatient Family Medicine (also commonly called primary care) after completing residency. This pathway is included to illustrate that primary care physicians in the U.S. may be internal medicine or family medicine physicians

In the U.S., Internal Medicine (IM) residency training, which lasts 3 years after graduation from medical school (called post-graduate years [PGY] 1, 2 and 3), is a required first step on the path to physician careers in general IM or in medicine subspecialties. (Fig. 1) IM residency-trained physicians who do not seek subspecialty training after residency may practice general outpatient IM (also called primary care), general inpatient IM (also called hospitalist medicine), or a combination of both. Published literature reveals several programs within IM residencies to provide training in underserved medicine (UM), including Montefiore Medical Center’s Residency Program in Social Medicine [11], University of California Davis’s Transforming Education and Community Health Program [12], Johns Hopkins University’s Urban Health Primary Care Residency Track [13], and Beth Israel Deaconess Medical Center’s HIV Primary Care Track [14], however most seek to produce PCPs. The University of Alabama at Birmingham published early information on its Health Disparities Track, which was available to all IM residents, however their small sample size limited program evaluation [15].

We describe the Underserved Medicine and Public Health (UMPH) program, a two-year longitudinal UM curriculum at the George Washington University internal medicine (GWIM) residency program. The overall goal of UMPH is to nurture and retain resident interest in caring for underserved patients regardless of their chosen field, thereby building a pipeline to create mission-minded medicine subspecialty physicians skilled at and dedicated to caring for underserved patients.

## Methods

### Setting and participants

GWIM residency program has 93 residents (31 per PGY) who predominantly train at an urban university hospital and a Veterans Affairs medical center. As part of training, all residents are required to see patients longitudinally in the outpatient general IM clinic at our academic practice. We use a 4 + 1 schedule [16], which means residents have 4 weeks of a clinical assignment (usually inpatient) followed by one recurring “+ 1” outpatient week. The “+ 1” week includes five outpatient general IM clinic sessions, two subspecialty clinic sessions and educational conferences. This pattern is repeated every 5 weeks across the 3 years of residency. Typically, about two-thirds of GWIM residency program graduates pursue subspecialty fellowship training and one-third choose to practice general IM.

We invite all new GWIM residents to apply to UMPH via email. Applications are reviewed by faculty. Participants are selected based on history of work with vulnerable populations and likelihood to pursue a career with the underserved; preference is given to residents from backgrounds underrepresented in medicine, those who speak Spanish and to residents with an expressed preference for non-primary care careers.

### Description of intervention

UMPH is a two-year program that begins at the beginning of PGY2 year. It has three components [1]: placement at a CHC with a community physician preceptor [2], evening seminars and facilitated reflection assignments, and [3] a practicum project. We schedule all UMPH activities in “+ 1” weeks; UMPH residents go to their assigned CHC in lieu of the two subspecialty clinic sessions. Over the course of PGY2 and 3 years, UMPH residents complete 40 clinic sessions at a CHC, ten evening seminars and ten facilitated reflection activities. Evening seminars and facilitated reflection assignments include topics such as health disparities, homeless health, health literacy, HIV, harm reduction, advocacy, promoting behavior change, medical-legal partnership and criminal justice health. (See Appendix for syllabus.) Topics were chosen after literature review and discussion between local faculty and community physician experts and were improved iteratively incorporating feedback from participants.

Originally, we planned for four participants per year. Due to increased interest, we expanded to a maximum of seven residents. UMPH began in 2013 and has 29 graduates.

### Program evaluation

In September 2020, we sent an anonymous survey link via email to all 29 graduates of the first 7 years of UMPH using SurveyMonkey (SurveyMonkey Inc., San Mateo, California). We sent two reminder emails to nonrespondents, each 2 weeks apart, beginning 2 weeks after the first invitation email. Email addresses used were those given to our program at the time of graduation; two graduates had emails that bounced.

The survey consisted of 27 questions using a 4-point Likert scale with no neutral option (strongly disagree, disagree, agree, strongly agree) to assess UMPH overall as well as perceived impact on attitudes, skills, and career choices; eight questions assessing individual components of UMPH; eight questions querying demographic and career information, and five open-ended questions regarding career motivations and suggestions for improvement. We used residency program records to determine whether graduates entered subspecialty training after completing residency.

Data were analyzed using descriptive statistics (frequencies) using SurveyMonkey. This study was declared exempt by the Institutional Review Board of The George Washington University (#190928).

## Results

Seventeen graduates responded to our survey (17/29, 59%). Two did not complete the entire survey, including demographic questions. Forty-seven percent (7/15) were female, 60% (9/15) identified as White/Caucasian, 13% (2/15) as Black/African American, 33% (5/15) as Hispanic/Latino, and 13% (2/15) as Asian/Pacific Islander.

UMPH appears to have achieved its goals. Ninety-four percent of respondents (16/17) strongly agreed that UMPH “exposed me to important topics in underserved medicine,” and “provided a meaningful forum to discuss important issues in underserved medicine,” and 93% (14/15) strongly agreed that UMPH “was an effective use of my time.” Eighty-eight percent (15/17) strongly agreed that UMPH helped foster their interest in treating specific underserved populations. All either strongly agreed (71%, 12/17) or agreed (29%, 5/17) that “UMPH provided me with a sense of belonging to a community of healthcare providers with a commitment to the underserved.” All either strongly agreed (53%, 9/17) or agreed (47%, 8/17) that “I provide better care today to my patients because of UMPH.” (Table 1).

**Table 1:** Survey responses by graduates of the Underserved Medicine and Public Health program, surveyed September 2020. (*n* = 17)

Question	Responses (n = 17) Number (%)			
	Strongly Disagree	Disagree	Agree	Strongly Agree
The UMPH program…				
…exposed me to important topics in public health.	0	0	2 (12)	15 (88)
…exposed me to important topics in underserved medicine.	0	0	1 (6)	16 (94)
…provided a meaningful forum to discuss important issues in public health.	0	0	2 (12)	15 (88)
…provided a meaningful forum to discuss important issues in underserved medicine.	0	0	1 (6)	16 (94)
…broadened my knowledge of career options in medicine and public health.	0	2 (12)	7 (41)	8 (47)
… improved my ability to analyze public health issues.	0	1 (6)	10 (59)	6 (35)
…provided clinical exposure to underserved populations in the Washington, DC area.	0	0	2 (12)	15 (88)
…helped foster my interested in treating specific underserved populations.	0	0	2 (12)	15 (88)
My understanding of key public health concepts improved as a result of UMPH.	0	0	11 (65)	6 (35)
In my daily work today, I use concepts or skills that were fostered by UMPH.	0	1 (6)	9 (53)	7 (41)
I provide better care today to my patients because of UMPH.	0	0	8 (47)	9 (53)
I have more interest in a career that involves public health as a result of UMPH.	0	1 (6)	11 (65)	5 (29)
I have more interest in a career that involves underserved populations as a result of UMPH.	0	2 (12)	5 (29)	10 (59)
I have more interest in a career that involves being a physician-leader as a result of UMPH.	0	3 (18)	7 (41)	7 (41)
I value interdisciplinary healthcare team members more highly because of UMPH.	0	2 (12)	6 (35)	9 (53)
UMPH provided me with a sense of belonging to a community of healthcare providers with a commitment to the underserved.	0	0	5 (29)	12 (71)
UMPH helped me meet my career goals.	0	0	8 (47)	9 (53)
UMPH allowed me to gain confidence in my ability to provide healthcare to underserved patients.	0	1 (6)	6 (35)	10 (59)
UMPH allowed me to gain comfort providing healthcare to underserved patients.	0	0	5 (29)	12 (71)
UMPH allowed me to enjoy providing healthcare to underserved patients.	0	0	3 (18)	14 (82)
Physicians have an important role in identifying social determinants of health for their patients.	0	1 (6)	2 (12)	14 (82)
I would recommend UMPH to current residents.	0	0	2 (12)	15 (88)
UMPH made me more likely to practice primary care.	0	11 (65)	3 (18)	3 (18)
UMPH should target residents pursuing primary care as a career pathway.	0	10 (59)	5 (29)	2 (12)
UMPH should target residents pursuing subspecialty care as a career pathway.	0	7 (41)	10 (59)	0
Providing healthcare to the underserved is fulfilling for me.	0	0	3 (18)	14 (82)
Overall, the UMPH experience was an effective use of my time.^a^	0	0	1 (7)	14 (93)

^a^ Two respondents did not complete the survey; for this question *n* = 15

UMPH seemingly achieved its goals even for residents choosing subspecialty careers. Forty-eight percent of UMPH graduates (14/29) pursued subspecialty training after residency, including infectious diseases (6 graduates), cardiology [3], hematology/oncology [2], nephrology [1] rheumatology [1] and geriatrics [1]. All 8 respondents who entered subspecialty training said that their decision to do so was not influenced by UMPH. Among UMPH graduates who did not pursue subspecialty training, ten of 15 practice predominantly inpatient general IM (hospitalist medicine). Nearly two-thirds of respondents (65%, 11/17) disagreed with the statement “UMPH made me more likely to practice primary care.” Fifty-nine percent agreed that “UMPH should target residents pursuing subspecialty care as a career pathway” and disagreed that “UMPH should target residents pursuing primary care as a career pathway.”

Because residents choose to apply to UMPH, we seek to nurture and retain baseline interest in caring for underserved patients. Although 88% (15/17) of respondents agreed or strongly agreed that they have more interest in a career that involves underserved populations as a result of UMPH; 12% (2/17) disagreed. It is unclear whether disagreement reflects decreased interest, lack of increase, or an endorsement that pre-existing strong interest was unrelated to UMPH.

## Discussion

The U.S. has a present and growing need for access to subspecialty physicians for patients from underserved communities. We believe that programs like UMPH can nurture and retain interest in caring for underserved patients and provide trainees with skills needed to do so regardless of planned subspecialty field. Survey responses from participants up to 5 years after graduation suggest that the program is successful at achieving its goals. It remains unknown whether graduates will continue to care for underserved patients throughout their careers. Areas for further study include sustainability, whether UM training results in improved subspecialty access for underserved patients, and whether subspecialists face different barriers than PCPs when caring for underserved patients.

These data are limited by our 59% survey response rate. They describe the effectiveness of UMPH but are necessarily descriptive rather than comparative to non-participants. Selection bias is unavoidable -- those who applied and were selected for participation had experience working with undeserved patients, expressed interest in caring for the underserved, and were motivated to complete additional activities as a supplement to residency training. These data cannot be extrapolated to all IM residents.

Implementing UMPH taught us several lessons. We had previously designed a two-week community health curriculum [17], allowing us to build relationships with local CHCs and providers, and to receive feedback on topics of greatest interest to our residents. This helped us to transition to UMPH. After evening seminars and reflection assignments were designed, faculty time was needed to implement ten two-hour evening seminars per year (five for PGY2s and five for PGY3s) and to coordinate clinic schedules. We found having two faculty co-directors made this easier. We have found a number of mission-consistent community partners who have been interested in teaching in the program as clinic preceptors or guest speakers for evening seminars. We cannot offer financial incentives, however we offer precepting physicians voluntary faculty appointments, which grant access to our institution’s library and a university identification card.

Other IM residency programs have written that scheduling constraints are a major barrier to incorporating social determinants of health curricula into residency, limiting attendance and participation [18, 19]. This was true when we launched UMPH, however putting all UMPH activities in “+ 1” weeks, and coordinating UMPH residents’ schedules such that their “+ 1″ weeks were aligned worked well for coordinating evening seminars and facilitated reflection assignments. It also addressed the concerns of residents planning to apply for subspecialty training after residency, by allowing for UMPH participation without decreasing available time for research and subspecialty rotations, which typically occur during our “4″ week blocks. The downside of this schedule is that participants’ community-based clinic activities occur during the same week, requiring more community sites to accommodate them contemporaneously. We offset the PGY2 and PGY3 “+ 1 weeks” from each other, which helped. Community clinic sites’ schedule restrictions required us to have flexibility in the timing of our residents’ work in the university outpatient general IM clinic. UMPH co-directors were university clinic supervisors and were motivated to help accommodate scheduling impacts.

## Conclusion

We hope that IM residency programs consider offering intensive training opportunities focused on caring for underserved patients not only for future PCPs, but for all interested trainees. Financial and institutional barriers may continue to impede subspecialty access beyond provider-level factors. Future curricula should include training in skills needed to advocate for policy change locally or nationally to most effectively decrease barriers to accessing subspecialty care.

## Data Availability

The datasets used during the current study are available from the corresponding author on reasonable request.

## Appendix

### Sample Underserved Medicine and Public Health (UMPH) Program Syllabus

#### Program Description & Objectives

The Underserved Medicine and Public Health (UMPH) Program is a two-year training program for residents who have a passion for serving the underserved. It is designed to supplement Internal Medicine Residency training at the George Washington University.

The overall goals of UMPH are to:

1. Enhance resident competency in providing healthcare to underserved patients;

2. Deepen resident knowledge and skills in public health and underserved medicine;

3. Develop public health and underserved medicine leadership potential.

By the completion of the program, UMPH Scholars should achieve the following learning objectives:

1. Create competent, compassionate and team-based medical outpatient care plans for patients in underserved community healthcare settings.

2. Identify career opportunities, management strategies, and challenges for physicians with underserved medicine and public health interests.

3. Design and execute a practicum project addressing a key public health or underserved medicine issue.

The UMPH program has three main components [1]: community clinic [2], evening seminars and reflection assignments, and [3] a practicum project.

I. Community Clinic

Each UMPH Scholar is assigned to one Community Clinic, where you will see patients with a regular preceptor two sessions per + 1 ambulatory week as a “second continuity clinic.” The Community Clinic experience provides you with 1) the opportunity to provide longitudinal care in a unique community-based setting and 2) the opportunity for mentorship with an experienced community provider. Over time, we hope you will develop a mini patient panel at your Community Clinic (much like you have at your residency continuity clinic).

It is your responsibility to let your preceptor know if you have any schedule changes that affect your community clinic as far in advance as possible. Likewise, establish a communication protocol with your preceptor for days where your preceptor may have vacation or other absences.

II. Evening Seminars and Reflection Assignments

The UMPH concentration includes evening seminars and reflection assignments. This curriculum provides you with the opportunity to build your knowledge base in public health as well as the opportunity to reflect on your clinical experiences from a population-based perspective. Each unit consists of Assigned readings to be completed independently, evening seminar discussion and possibly an application activity before the seminar.

Evening seminars will be held on Thursday evenings during your + 1 ambulatory week and will include dinner. Sometimes we will have a guest with special expertise join us. The purpose of reflection assignments is to allow you to build your knowledge base and to provide a space for personal reflection as well as a forum for group discussion via email. Participation is mandatory, as we will all get more out of it when all voices are heard!

**Table:**

Type of session	Topic
**PGY2**	
Evening Seminar	Orientation
Reflection Assignment	Health Disparities
Evening Seminar	Homeless Health with guest speaker
Reflection Assignment	Health Literacy
Reflection Assignment	(NA: Do the reading for our next session)
Evening Seminar	HIV & Outbreak Investigation with guest speaker
Reflection Assignment	Improving Access: Legal and Ethical Challenges
Evening Seminar	Practicum project brainstorming session
Reflection Assignment	Promoting Behavior Change and Medication Adherence
Evening Seminar	Harm Reduction: Field trip to community organization
**PGY3**	
Evening Seminar	Physicians as Advocates with guest speaker
Reflection Assignment	Advocacy in action!
Evening Seminar	Medical-Legal Partnership with guest speaker
Evening Seminar	Practicum project updates and brainstorming
Reflection Assignment	Physician Self-care
Evening Seminar	Criminal Justice Health
Reflection Assignment	(NA: Work on practicum project)
Evening Seminar	Human Trafficking
Reflection Assignment	Your choice – pick topic/reading as a group
Lunch presentation	Practicum Project presentations and UMPH graduation lunch

III. Practicum Project

Each UMPH scholar will design a practicum project during PGY2 year and carry it out during PGY3 year. You may work individually or in groups. We hope that you will take this opportunity to design a project that not only fits within the themes of UMPH, but also allows you to explore your own interests. For example, you may be interested in needs assessment, healthcare quality improvement, patient education, community outreach, advocacy, curriculum development, etc. You will be provided with an “UMPH Practicum Guide” with more details to guide your experience.

During an evening seminar of PGY2 we will have a workshop in which you will pitch your practicum idea to your colleagues, and we will brainstorm as a group to provide feedback and improve your project. Our last meeting of PGY3 year will be used present your practicum project!

## Abbreviations

HPSA: Health Professional Shortage Areas
PCP: Primary care physician
FQHC: Federally Qualified Health Center
CHC: Community health center
IM: Internal medicine
UM: Underserved medicine
UMPH: Underserved Medicine and Public Health
GWIM: George Washington University internal medicine
PGY: Post-graduate year
